# SEX-DIMORPHISM IN THE GENOMIC REGULATION OF AGING

**DOI:** 10.1093/geroni/igz038.2825

**Published:** 2019-11-08

**Authors:** Bérénice A Benayoun, Ryan A Lu, Nirmal K Sampathkumar

**Affiliations:** 1 University of Southern California, Leonard Davis School of Gerontology, Los Angeles, California, United States; 2 University of Southern California, Los Angeles, California, United States

## Abstract

The current cohort of human supercentenarians reveals a surprising predictor for achieving such an exceptional longevity: being female. Indeed, out of 34 living supercentenarians, 33 are women. We obtained samples from 4 and 20 months old female and male mice. Our data indicates that cytokine levels are differentially regulated with age in males vs. females, with pro-inflammatory cytokines specifically upregulated in the serum of old males, but not females. Because of the central role of macrophages in inflammation and their infiltration in tissues with age, we have generated RNA-seq from purified macrophages of aging animals. Female macrophages displayed ~7-20-fold more transcriptional remodeling with aging than males. Pathways specifically downregulated in females with aging included lysosome, inflammation and phagolysosome. Consistently, our data shows that aged female, but not male macrophages, display decreased phagocytic efficiency. Our results support the notion that there are differences in aging trajectories in female vs. male mice.

